# Investigation of the Molecular Mechanism of Coagulopathy in Severe and Critical Patients With COVID-19

**DOI:** 10.3389/fimmu.2021.762782

**Published:** 2021-12-16

**Authors:** Daniel Elieh Ali Komi, Yaghoub Rahimi, Rahim Asghari, Reza Jafari, Javad Rasouli, Mehdi Mohebalizadeh, Ata Abbasi, Rahim Nejadrahim, Farzin Rezazadeh, Vahid Shafiei-Irannejad

**Affiliations:** ^1^ Cellular and Molecular Research Center, Cellular and Molecular Medicine Institute, Urmia University of Medical Sciences, Urmia, Iran; ^2^ Hematology, Immune Cell Therapy, and Stem Cells Transplantation Research Center, Clinical Research Institute, Urmia University of Medical Sciences, Urmia, Iran; ^3^ Nephrology and Kidney Transplant Research Center, Clinical Research Institute, Urmia University of Medical Sciences, Urmia, Iran; ^4^ Department of Epidemiology and Biostatistics, Faculty of Medicine, Urmia University of Medical Sciences, Urmia, Iran; ^5^ Student Research Committee, Urmia University of Medical Sciences, Urmia, Iran; ^6^ Department of Pathology, Faculty of Medicine, Urmia University of Medical Sciences, Urmia, Iran; ^7^ Department of Infectious Diseases, Faculty of Medicine, Urmia University of Medical Sciences, Urmia, Iran; ^8^ Department of Emergency Medicine, Faculty of Medicine, Urmia University of Medical Sciences, Urmia, Iran

**Keywords:** COVID-19, coagulopathy, fibrinogen, protein C (PC), protein S, antithrombin III (ATIII), D-dimer (DD)

## Abstract

Coagulopathy is a frequently reported finding in the pathology of coronavirus disease 2019 (COVID-19); however, the molecular mechanism, the involved coagulation factors, and the role of regulatory proteins in homeostasis are not fully investigated. We explored the dynamic changes of nine coagulation tests in patients and controls to propose a molecular mechanism for COVID-19-associated coagulopathy. Coagulation tests including prothrombin time (PT), partial thromboplastin time (PTT), fibrinogen (FIB), lupus anticoagulant (LAC), proteins C and S, antithrombin III (ATIII), D-dimer, and fibrin degradation products (FDPs) were performed on plasma collected from 105 individuals (35 critical patients, 35 severe patients, and 35 healthy controls). There was a statically significant difference when the results of the critical (CRT) and/or severe (SVR) group for the following tests were compared to the control (CRL) group: PT_CRT_ (15.014) and PT_SVR_ (13.846) (PT_CRL_ = 13.383, *p* < 0.001), PTT_CRT_ (42.923) and PTT_SVR_ (37.8) (PTT_CRL_ = 36.494, *p* < 0.001), LAC_CRT_ (49.414) and LAC_SVR_ (47.046) (LAC_CRL_ = 40.763, *p* < 0.001), FIB_CRT_ (537.66) and FIB_SVR_ (480.29) (FIB_CRL_ = 283.57, *p* < 0.001), ProC_CRT_ (85.57%) and ProC_SVR_ (99.34%) (ProC_CRL_ = 94.31%, *p* = 0.04), ProS_CRT_ (62.91%) and ProS_SVR_ (65.06%) (ProS_CRL_ = 75.03%, *p* < 0.001), D-dimer (*p* < 0.0001, *χ* ^2^ = 34.812), and FDP (*p* < 0.002, *χ* ^2^ = 15.205). No significant association was found in the ATIII results in groups (ATIII_CRT_ = 95.71% and ATIII_SVR_ = 99.63%; ATIII_CRL_ = 98.74%, *p* = 0.321). D-dimer, FIB, PT, PTT, LAC, protein S, FDP, and protein C (ordered according to *p*-values) have significance in the prognosis of patients. Disruptions in homeostasis in protein C (and S), VIII/VIIIa and V/Va axes, probably play a role in COVID-19-associated coagulopathy.

## Introduction

**Figure 1:**
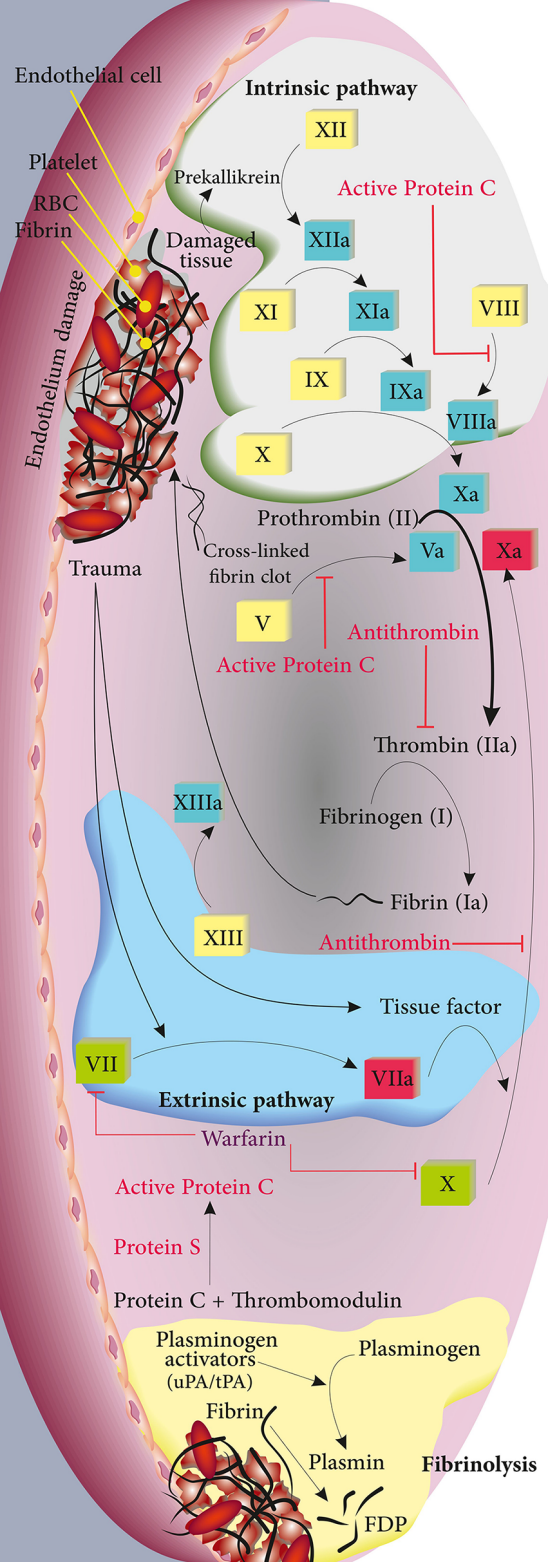
Depiction of the intrinsic, extrinsic, and fibrinolysis pathways in *green*, *blue*, and *yellow*, respectively. The involved coagulation factors for each pathway are shown. Regulatory proteins and their target molecules are shown in *red*.

Coagulation is a dynamic process that is driven by the regulated proteolytic activation of zymogens (commonly known as coagulation factors) in injured vessels. Coagulation factors, except for FVIII, which is produced by liver sinusoidal endothelial cells and lymphatic tissue, are all produced by hepatocytes (1). The main mechanisms (pathways) that trigger blood clotting include intrinsic and extrinsic pathways, each including a set of coagulation proteins in which factors I, II, IX, X, XI, and XII are the main factors in the intrinsic pathway and factors I, II, VII, and X are the factors described in the extrinsic pathway. Activated partial thromboplastin time (aPTT) and prothrombin time (PT) tests primarily measure the activity of the factors involved in the intrinsic and extrinsic pathways, respectively (2, 3). Moreover, the common pathway is composed of factors I, II, V, VIII, and X (4). The proper proteolytic activation of coagulation factors controlled by a variety of regulatory proteins results in the conversion of soluble fibrinogen to insoluble fibrin strands (5). Fibrinogen, a 340-kDa glycoprotein, is an acute-phase protein consisting of three polypeptide chains, Aα, Bβ, and γ, and becomes upregulated in response to injury and inflammation (5). The term lupus anticoagulant (LAC) is used to determine heterogeneous immunoglobulins, their function resulting in the inhibition of phospholipid-dependent coagulation reactions (6). Moreover, LACs can prolong the PTT test; therefore, a LAC test is used to evaluate prolonged PTT (7). Coagulation regulatory proteins such as antithrombin III (ATIII), protein C, and D-dimer are involved in the normal function and homeostasis of the coagulation system. ATIII, a crucial anticoagulant molecule in mammalian blood, benefits from its cofactor, heparin, to inhibit the coagulation proteases, mainly thrombin and factor Xa (8). Proteins C and S are vitamin K-dependent glycoproteins. Protein S, the cofactor for protein C, supports the activated protein C in the presence of phospholipids and calcium in the inactivation of membrane-bound factors V (FVa) and FVIIIa (9). The mechanistic pathways through which protein C exerts its effects on the coagulation cascades include degrading factors V/Va and VIII/VIIIa, releasing a tissue-type plasminogen activator, and stimulating fibrinolysis by interacting with the plasminogen activator inhibitor (10). Fibrinolysis is an essential step in homeostasis that is finely controlled by a set of cofactors and inhibitors. Plasmin acts as the primary fibrinolysin and is activated from plasminogen in the presence of a tissue plasminogen activator (tPA) or urokinase (uPA) (11). Plasmin, after being produced, lyses the cross-linked fibrin polymers and consequently forms fibrin degradation products (FDPs) such as D-dimer, which is widely used as a specific marker for thrombosis and physiological fibrinolysis (12) (**Figure 1**). The coagulopathy and abnormal results in coagulation tests have become common features reported in patients with COVID-19 from the very early days of the emergence of the new coronavirus strain. We listed both the common coagulation tests, including PT, PTT, fibrinogen, and D-dimer, and those rarely investigated, such as regulatory proteins C and S as well as ATIII, in patients with COVID-19 along with the main results in **Table 1**. COVID-19-dependent coagulopathy gained attention when PT, aPTT, fibrinogen, and D-dimer tests were recommended by researchers to evaluate the proper homeostasis of the system associated with the prognosis of patients. Moreover, the prophylactic use of anticoagulants was proven to be effective in lowering the mortality rate and highlighted the role of the coagulation system in COVID-19 (31). The link between thrombosis and COVID-19 as an inflammatory disease has been investigated (32, 33). In the present study, we used a coagulation panel of nine coagulation tests to assess the coagulation pathways in 105 included individuals to determine the molecular mechanism through which COVID-19 disrupts the homeostasis of the coagulation system.

**Table 1:** Brief literature review of the clinical laboratory coagulation indexes previously reported in coronavirus disease 2019 (COVID-19) patients.

Test (unit)	Target population or center/country	Consistency (with our results)	Included patients/main findings	Reference
PT (s)	Tianyou Hospital, Wuhan, China	✓	115 patients were included. The mean ± SD results for the PT test in critical and severe groups were 13.70 ± 3.38 and 12.14 ± 1.16 s, respectively.	(13)
	Jinyintan Hospital and Wuhan Pulmonary Hospital, China	✓	191 patients [survivors (*n* = 137), non-survivors (*n* = 54)] were studied. PT_Survivors_ = 11.4 s, PT_non-Survivors_ = 12.1 s (*p* = 0·0004) 7 (13%) of expired patients had PT ≥ 16, while only 4 (3%) of patients who survived had PT ≥ 16.	(14)
	Chongqing, China	✓	135 patients were recruited. The mean PT values for the critical and severe groups were 11.3 and 10.8 s, respectively (*p* = 0.011)	(15)
aPTT (s)	Tongji Hospital, Wuhan, China	✓	147 patients were enrolled. The mean aPTT results were 42.4 in expired patients(*N* = 35) and 40.6 in survivors (*N* = 112, *p* = 0.256)	(16)
	Tianyou Hospital, Wuhan, China	✓	115 patients were included. Critical group had higher aPTT results when compared with the severe and mild groups (36.98 ± 8.60, 36.47 ± 9.29, and 34.9 ± 9.17 s, respectively).	(13)
	Chongqing, China	✓	135 patients were recruited. The mean aPTT values for the critical and severe groups were 29.7 and 26.6 s, respectively (*p* = 0.011).	(15)
Fibrinogen	Renmin Hospital of Wuhan University	✓	94 patients were studied. 502 ± 153 mg/ml in COVID-19 patients compared to 290 ± 53 mg/ml in the control group (*p* < 0.001)	(17)
	Suzhou Hospital	✓	75 patients were enrolled. While the average fibrinogen levels in controls were 200–400 mg/ml, patients were reported to have significantly higher levels (430 ± 119 mg/ml, *p* < 0.05).	(18)
	Fuyang Second People’s Hospital	✓	43 patients were studied. The average fibrinogen levels in the severe group was 384 ± 100 mg/ml and in the mild group was 311 ± 083 mg/ml (*p* = 0.14).	(15)
Anti-lupus coagulant (s)	Hospitals in Liechtenstein and Switzerland	N/A	64 patients were studied. Higher total IgA and IgA anti-phospholipid antibodies were found in severe patients (*p* < 0.001).	(19)
	Lariboisière Hospital, Paris	✓	74 consecutive mechanically ventilated patients were enrolled. LAC was positive in 63 patients (85%). 23 out of 28 patients with thrombotic complications were positive.	(20)
	Tan Tock Seng Hospital, Singapore	✓	12 ICU patients with severe COVID-19 pneumonia were included. Lupus anticoagulants were present in 50% of patients.	(21)
Protein C (%)	R Adams Cowley Shock Trauma Center, Maryland, USA		10 critically ill patients were included, who were using mechanical lung ventilation. The mean protein C activity was 104 ± 40 (normal = 83%–168%).	(22)
	Tenon University Hospital, Paris, France		430 patients were included. Protein C activity was higher in conventional (mild) patients (97%, 79–113) than the worsening disease group (88%, 71–100).	(23)
	Tan Tock Seng Hospital, Singapore		12 ICU patients with severe COVID-19 pneumonia were included. The average activity of protein C was 77.5%.	(21)
Protein S (%)	Colentina University Hospital Bucharest, Romania	✓	91 patients were enrolled, of whom 21 (23.3%) died. 65% of the patients were reported to have decreased protein S activity. Death cases had lower protein S activity (median = 42% *vs.* 58%, *p* < 0.001).	(24)
	Tan Tock Seng Hospital, Singapore	✓	12 ICU patients with severe COVID-19 pneumonia were included. The average activity of protein S was 65.2%.	(21)
	Gregorio Marañon Hospital, Madrid, Spain	N/A	206 patients were enrolled. The average protein S activity in COVID-19 patients with thrombosis was 60.9 (46.3–69.4), while the mean activity in patients without thrombosis was 53.2 (42.1–66.9, *p* = 0.429).	(25)
ATIII (%)	Milan, Italy	✓	24 intubated patients were included. The mean antithrombin activity was slightly decreased [74 U/dl, reference range mean = 102 (82–122)].	(26)
	Tan Tock Seng Hospital, Singapore	✓	12 ICU patients with severe COVID-19 pneumonia were included. The average activity of ATIII was 84.4%.	(21)
	R Adams Cowley Shock Trauma Center, Maryland, USA	✓	10 critically ill patients were included, who were using mechanical lung ventilation. The mean ATIII activity was 84 (normal = 75%–135%).	(22)
D-dimer (μg/ml)	Tianyou Hospital of Wuhan, China	N/A	Classified 115 patients into four groups according to the disease severity. 18 out of 22 deceased patients had increased levels of D-dimer in the first lab test (3.47 ± 7.41 mg/l in expired patients compared to 0.87 ± 1.73 mg/l in discharged patients). The change in CT imaging was in correlation with the increase of the D-dimer levels.	(13)
	Jin Yin-tan Hospital, Wuhan, China	✓	41 patients were included (ICU patients: *n* = 13; no ICU care: *n* = 28). D-dimer levels on admission were higher in ICU patients (2.4 mg/L) than those in non-ICU patients (0.5 mg/L).	(27)
	Texas, USA	N/A	15,313 hospitalized patients >18 years old 285 were reported to have acute ischemic stroke, who had higher D-dimer levels at admission (1.42 *vs.* 0.94 µg/ml FEU, *p* < 0.001). Elevated D-dimer levels >5.15 µg/ml was shown to increase mortality nearly 3 times.	(28)
	Tianyou, Puren, and China Resources & WISCO General Hospitals, Wuhan, China	✓	1,114 patients with COVID-19 were included. The value 2.025 mg/L was determined as the optimal probability cutoff to predict death. The D-dimer levels of the expired patients were notably higher than those of surviving individuals. D-dimer levels of COVID-19 patients with DIC were higher when compared to those without DIC.	(29)
FDP	Tongji Hospital, Wuhan	✓	147 patients were enrolled. The mean FDP levels were 70.8 in expired patients (*N* = 35) and 4.8 g/L in survivors (*N* = 112, *p* < 0.001).	(16)
	Renmin Hospital of Wuhan University	✓	94 patients were studied. 33.83 ± 82.28 mg/L in COVID-19 patients compared to 1.55 ± 1.09 mg/L in healthy controls (*p* < 0.001)	(17)
	Tongji Hospital of Huazhong University, Wuhan	✓	183 patients were included, among them 21 patients expired. Significant increase in FDP levels between survivors and non-survivors [4.0 (4.0–4.3) *vs.* 7.6 μg/ml (4.0–23.4)]	(30)

PT, prothrombin time; aPTT, activated partial thromboplastin time; ATIII, antithrombin III; FDP, fibrin degradation product; LAC, lupus anticoagulant; N/A, not applicable; FEU, fibrinogen equivalent unit; DIC, disseminated intravascular coagulation.

## Materials and Methods

### Inclusion/Exclusion Criteria

We followed the guidelines for Corona Virus Disease 2019 edited by the Iranian National Health Commission (similar to the WHO guidelines and the New Coronavirus Pneumonia Prevention and Control Program, 7th edition, published by the National Health Commission of China) to classify the patients into critical and severe groups (34, 35). The criteria used for the inclusion of individuals into each group are summarized in **Table 2**. All 70 included patients had a positive result of the nucleic acid test of severe acute respiratory syndrome coronavirus 2 (SARS-CoV-2) by RT-PCR using primers targeting the RNA-dependent RNA polymerase (RdRP) and either nucleocapsid (N), envelope (E), or spike (S) genes. A negative result (using the same probes) was used as the main inclusion criterion for the control (CRL) group. All patients were tested for lung involvement by CT imaging. Moreover, individuals in the CRL group had no physical features of COVID-19 such as fever or coughing and never had a positive RT-PCR result before. We also checked immediate family history to exclude those who have and/or had a family member with a positive PCR test to exclude the possibility of including asymptomatic carriers as healthy controls. Considering that almost all coagulation factors are produced in the liver, any functional disorder in the organ may result in abnormal plasma levels of the factors; therefore, we performed liver functional tests (LFTs) for all 105 included individuals.

**Table 2:** Inclusion criteria for the recruitment of individuals into the control (CRL), severe (SVR), and critical (CTL) groups.

Group	Criteria
Control^a^	Having a negative result for severe acute respiratory coronavirus 2 (SARS-CoV-2) by RT-PCR during the last 48 h (to exclude the chance of infection even at the earlier stage among enrolled healthy controls) No history of abnormal liver function tests (both direct and total bilirubin, SGOT, SGPT, ALKP) (considering that the majority of coagulation factors are produced in the liver, applying this criterion ensures no healthy control has a liver disease) No history of COVID-19 positivity reported from any immediate family member (to minimize the chance of getting infected from immediate family members during the time between PCR test and the time of blood collection. Additionally, it helps minimize the chance of being an asymptomatic carrier) Having no signs of fever, coughing, or other physical features of COVID-19 Having no history of hemorrhagic diseases in the past and present (any individuals with a history of recent hemorrhagic events such as a recent operation or menstruation in females were excluded to avoid impacts on the coagulation hemostasis) No history of heparin, low-molecular-weight heparins (LMWHs), and warfarin therapy
Severe^b^, ^d^	Having respiratory distress (respiratory rate ≥30 times/min) Oxygen saturation ≤93% Progression of lesion >50% within 24–48 h in lung CT imaging
Critical^c^, ^d^	Having respiratory failure and requiring mechanical ventilation Shock Organ failure Requiring ICU treatment

SGOT, aspartate aminotransferase; SGPT, alanine aminotransferase; ALKP, alkaline phosphatase; COVID-19, coronavirus disease 2019.

a Thirty-five healthy controls were recruited [15 females (42.9%) and 20 males (57.1%)]. The average age was 50.34 ± 20.84 years.

b Thirty-five severely ill hospitalized individuals were recruited [15 females (42.9%) and 20 males (57.1%)]. The average age was 50.91 ± 16.42 years. Blood samples were collected immediately after admission before any other therapeutic and medical interventions. In this group, 3 patients had cardiovascular disease, 9 had hypertension, 7 were found with diabetes, and 1 with pulmonary disease. Patients in this group were hospitalized in either Urmia General Hospital or Urmia Taleghani Hospital.

c Thirty-five critically ill individuals in ICU were recruited [15 females (42.9%) and 20 males (57.1%)]. The average age was 52.03 ± 15.06 years. Blood samples were collected immediately after admission before any other therapeutic and medical interventions. In this group, 9 patients had cardiovascular disease, 15 had hypertension, 5 were found with diabetes, 1 with kidney disease, and 2 with pulmonary disease. Patients in this group were hospitalized in either Urmia General Hospital or Urmia Taleghani Hospital.

d Patients with underlying diseases who were using metformin, glibenclamide, captopril, or losartan to control their chronic diseases. These drugs are not reported to have effects on coagulation factors.

### Demographic Features of Patients

In this case–control study, 105 individuals were included and classified into three groups (critical, severe, and control), each consisting of 35 individuals [20 males (57.1%) and 15 females (42.9%)]. The demographic data are presented in **Table 3**. The mean ages in the three groups were 52.03 (SD = 15.06), 50.91 (SD = 16.42), and 50.34 years (SD = 20.84), respectively. In the critical (CTL) group, the mean weight and height were 72.51 ± 14.75 kg and 163.26 ± 13.88 cm (BMI = 27.86 ± 9.35), while the same parameters in the severe group were measured at 79.14 ± 9.95 kg and 171.80 ± 7.50 cm (BMI = 26.83 ± 3.09), respectively. In the severe (SVR) group, 3 patients had cardiovascular disease, 9 had hypertension, 7 were found with diabetes, and one had pulmonary disease. Furthermore, in the CTL group, 9 had cardiovascular disease, 15 had hypertension, 5 were found with diabetes, one with kidney disease, and two with pulmonary disease. These patients were using metformin, glibenclamide, captopril, or losartan to control their chronic diseases, which have no effects on the coagulation factors. Due to the prophylactic guidelines for the administration of anticoagulation drugs to patients with poor health conditions, we collected the samples at admission before any medical intervention. Moreover, we checked for history of any drug use that could potentially interfere with our results by referring to medical insurance records and through collecting information using an enrollment form. Assuming an *α* value set at 0.05 (type I error), *β* at 0.10 (type II errors), a dropout rate of 5%, and considering the results of Gao et al. (15), the sample size was set at a minimum of 35 patients in each group to compare the differences between the means. Patients in the severe and critical groups were selected from hospitalized patients in COVID and ICU wards, respectively, from either Urmia General Hospital or Taleghani Hospital in Urmia, Iran.

**Table 3:** Demographic features of the patients in control (CRL), severe (SVR), and critical (CTL) groups.

Parameter	Group	No.	Mean	SD	*p*-value
Age (years)	CRL	35	50.34	20.84	0.92
	SVR	35	50.91	16.42	
	CTL	35	52.03	15.06	
Weight	CRL	0	–	–	0.031
	SVR	35	79.14	9.95	
	CTL	35	72.51	14.75	
Height	CRL	0	–	–	0.002
	SVR	35	171.80	7.50	
	CTL	35	163.26	13.88	
BMI (kg/m^2^)	CRL	0	–	–	0.539
	SVR	35	26.83	3.09	
	CTL	35	27.86	9.35	

### Sample Collection and Preparation

Approximately 1.8 ml of peripheral blood was collected into tubes containing 0.2 ml sodium citrate (3.2%). The tubes were immediately gently mixed and centrifuged (1,200 × *g*, 10 min), and the appearance of the plasma was checked to exclude icteric (abnormal function of the liver), lipemic (as a preclinical error especially in photometric assays) (36), and hemolyzed specimens (37) or tubes with micro-clots (38). Considering that prolonged storage of plasma specimens negatively affects the results of coagulation tests (39), we managed to perform this study at the peak of the fifth wave of the disease in Iran in order to include as many patients as possible. This strategy helped us collect all the required samples rapidly and to perform the coagulation tests quickly without freezing the plasma samples. All tests were run within 3 h after sample collection. According to the partially low stability of D-dimer in plasma (40), and using semi-quantitative kits to measure D-dimer and FDPs, we performed these tests before the other tests.

### Materials

The PT (NeoPTimal), aPTT (C.K. PREST), CaCL_2_ (0.025 M), fibrinogen (STA-Liquid Fib), anti-lupus coagulant, fibrinogen, protein C (STACLOT), protein S (STACLOT), antithrombin III (STACHROM), Owren–Koller, Desorb-U, D-dimer, and FDP kits were purchased from Stago Co., Asnières sur seine, France. All tests, except for the D-dimer and FDPs, were performed using the fully automated STA Compact® System (Diagnostica Stago, Asnières sur seine, France). We used semi-quantitative D-dimer and FDP kits (benefiting from latex particles coated with monoclonal antibodies to D-dimer or FDP, respectively). Moreover, Toshiba Alexion 16-slice (Toshiba, Japan) and GE BrightSpeed Elite 16 Slice (Chicago, IL, USA) were used for CT scans of the patient groups, and micPCR Biomolecular Systems (Upper Coomera, Australia) was used for the PCR testing of all individuals.

### Performing Coagulation Tests

After obtaining the samples, we checked them twice before and after putting them on the mixer to exclude any samples with visible signs of clotting or micro-clots. After 5 min of mixing the samples, they were centrifuged and the obtained plasma samples were analyzed for D-dimer and FDPs; then, the plasma samples were poured into conventional plastic tubes and loaded into the autoanalyzer. To perform semi-quantitative D-dimer and FDP tests, we used the glycine buffer to dilute each plasma sample in plastic test tubes. For the D-dimer test, we added 20 ml of reagent 1 (including ready-to-use latex particles coated with mouse anti-human D-dimer monoclonal antibody) to 20 ml of undiluted/diluted plasma samples of each individual, mixed gently, and assessed the agglutination. The interpretation of the results was done using the protocol provided in **Table 4**. A similar protocol was used for the FDP test, but with only two diluted concentrations/titers (1:2 and 1:8) assessed for agglutination according to the instruction of the manufacturer. We performed the rest of the tests using a fully automated STA Compact® System (Diagnostica Stago, France) in a duplicate manner. We did quality control testing for each run using STA-System Control N+P.

**Table 4:** Approximate diluted/undiluted concentrations and the titers for the D-dimer and fibrin degradation product (FDP) test.

Test sample/titers					Levels		
Undiluted	1:2	1:4	1:8	1:16			
D-dimer					D-dimer (μg/ml FEU)		
(−)						<0.5	
(+)	(−)				≥0.5		<1.0
(+)	(+)	(−)			≥1.0		<2.0
(+)	(+)	(+)	(−)		≥2.0		<4.0
(+)	(+)	(+)	(+)	(−)	≥4.0		<8.0
(+)	(+)	(+)	(+)	(+)		≥8.0	
FDP					FDP (μg/ml)		
			1:2	1:8
			(−)	(−)		<5.0	
			(+)	(−)	≥5.0		<20
			(+)	(+)		≥20	

(+): presence of agglutination; (−): no agglutination.

FEU, fibrinogen equivalent unit.

### Statistical Analysis

The data obtained from the autoanalyzer for PT, PTT, fibrinogen, lupus anticoagulant, proteins C and S, and ATIII (quantitative tests) and for D-dimer and FDPs (semi-quantitative tests) were reported. Statistical analysis was performed using SPSS (ver. 21; IBM, Armonk, NY, USA). To compare the groups, we used one-way ANOVA, Fisher’s exact test, and chi-square tests. A *p*-value <0.05 was considered to be statistically significant.

## Results

### Analysis of the Results of the Three Studied Groups

**Figure 2:**
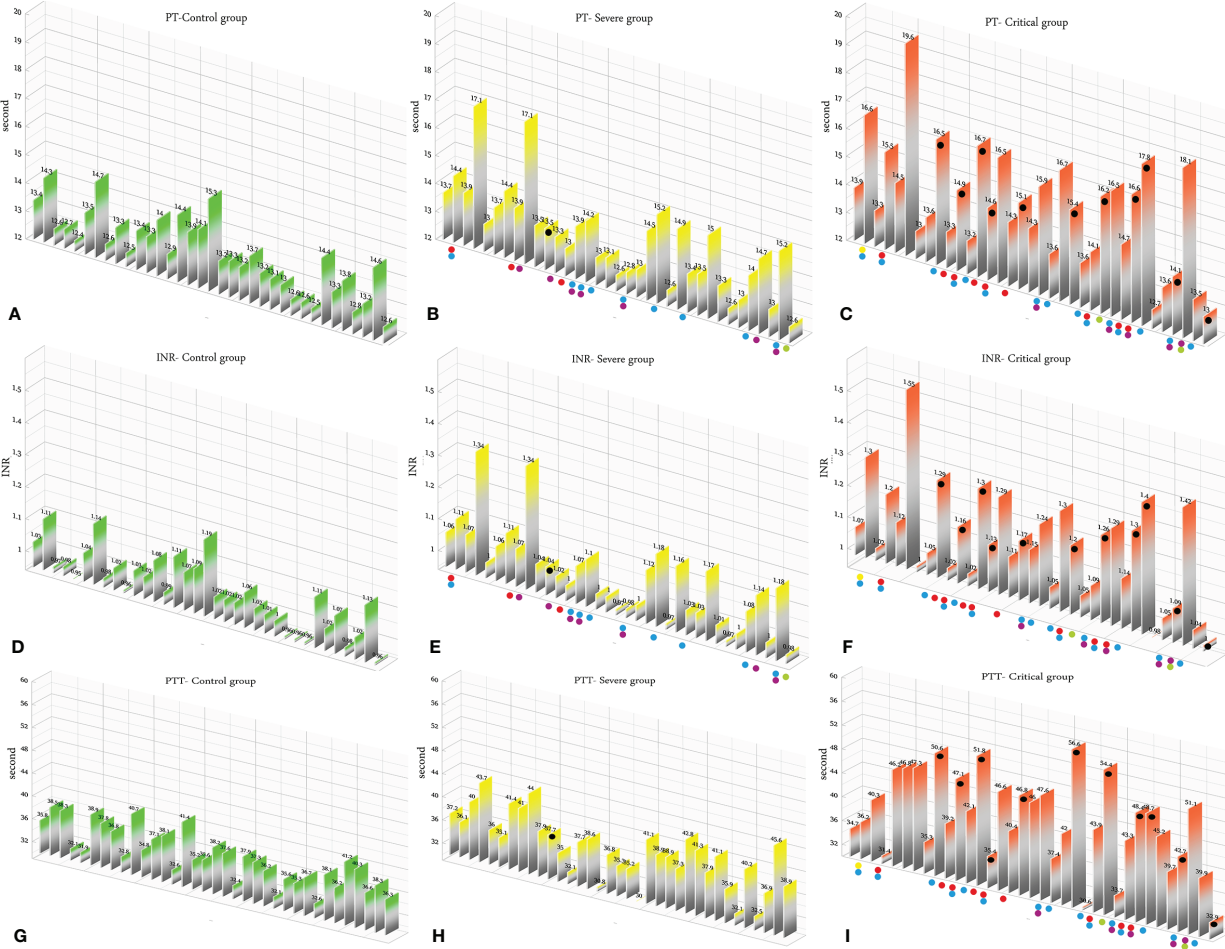
**(A–C)** Prothrombin time (PT) test results in the control (CRL, *green*), severe (SVR, *yellow*), and critical (CTL, *red*) groups. **(D–F)** International normalized ratio (INR) results in the CRL, SVR, and CTL groups. **(G–I)** Partial thromboplastin time (PTT) test results in the CRL, SVR, and CTL groups. Expired individuals in the SVR and CTL groups are shown with a *black bullet point*. *Bullet points* in *red*, *blue*, *purple*, *green*, and *yellow* indicate cardiovascular disease, hypertension, diabetes, pulmonary disease, and kidney disease, respectively, in the SVR and CTL groups.

**Figure 3:**
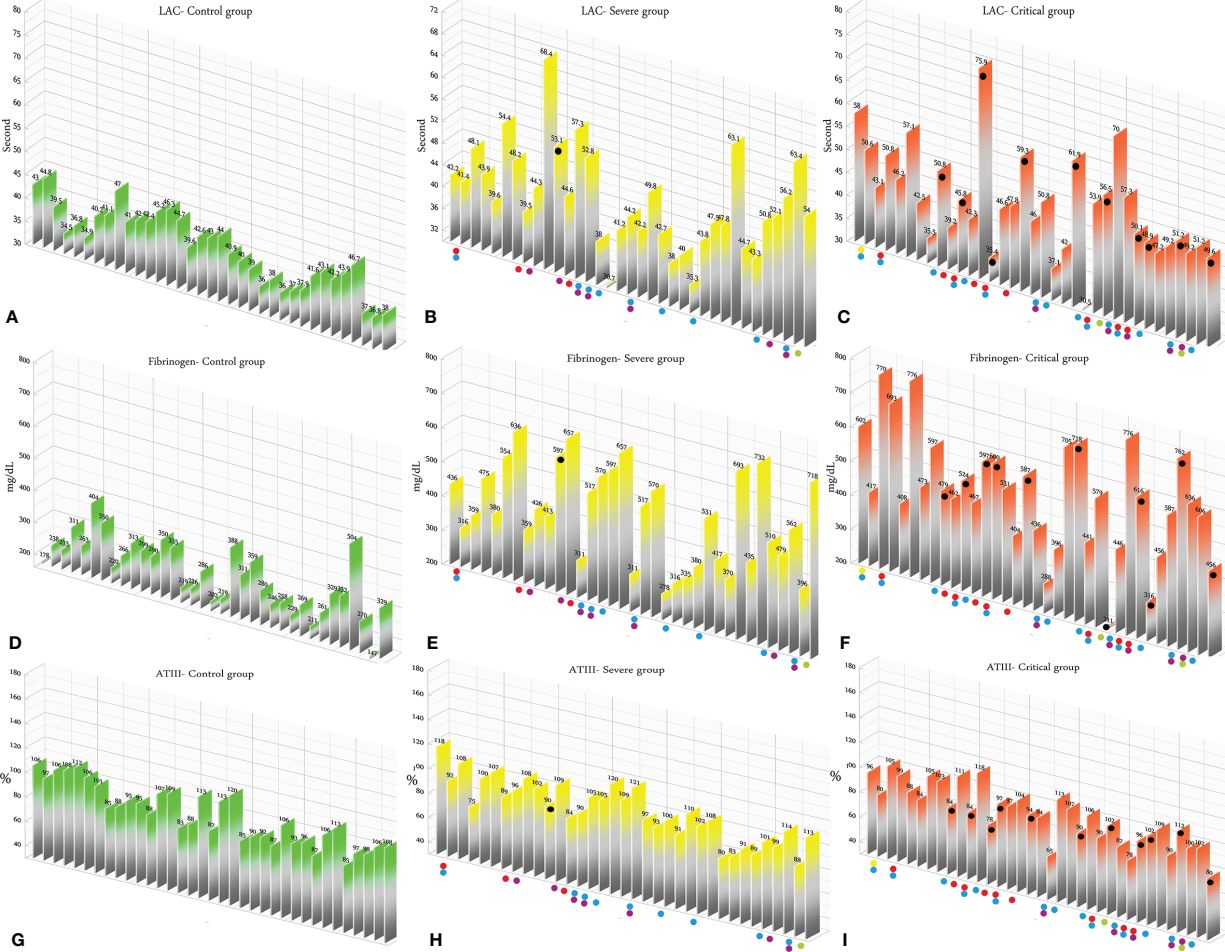
**(A–C)** Lupus anticoagulant test results in the control (CRL, *green*), severe (SVR, *yellow*), and critical (CTL, *red*) groups. **(D–F)** Fibrinogen results in the CRL, SVR, and CTL groups. **(G–I)** Antithrombin III (ATIII) test results in the CRL, SVR, and CTL groups. Expired individuals in the SVR and CTL groups are shown with a *black bullet point*. *Bullet points* in *red*, *blue*, *purple*, *green*, and *yellow* indicate cardiovascular disease, hypertension, diabetes, pulmonary disease, and kidney disease, respectively, in the SVR and CTL groups.

**Figure 4:**
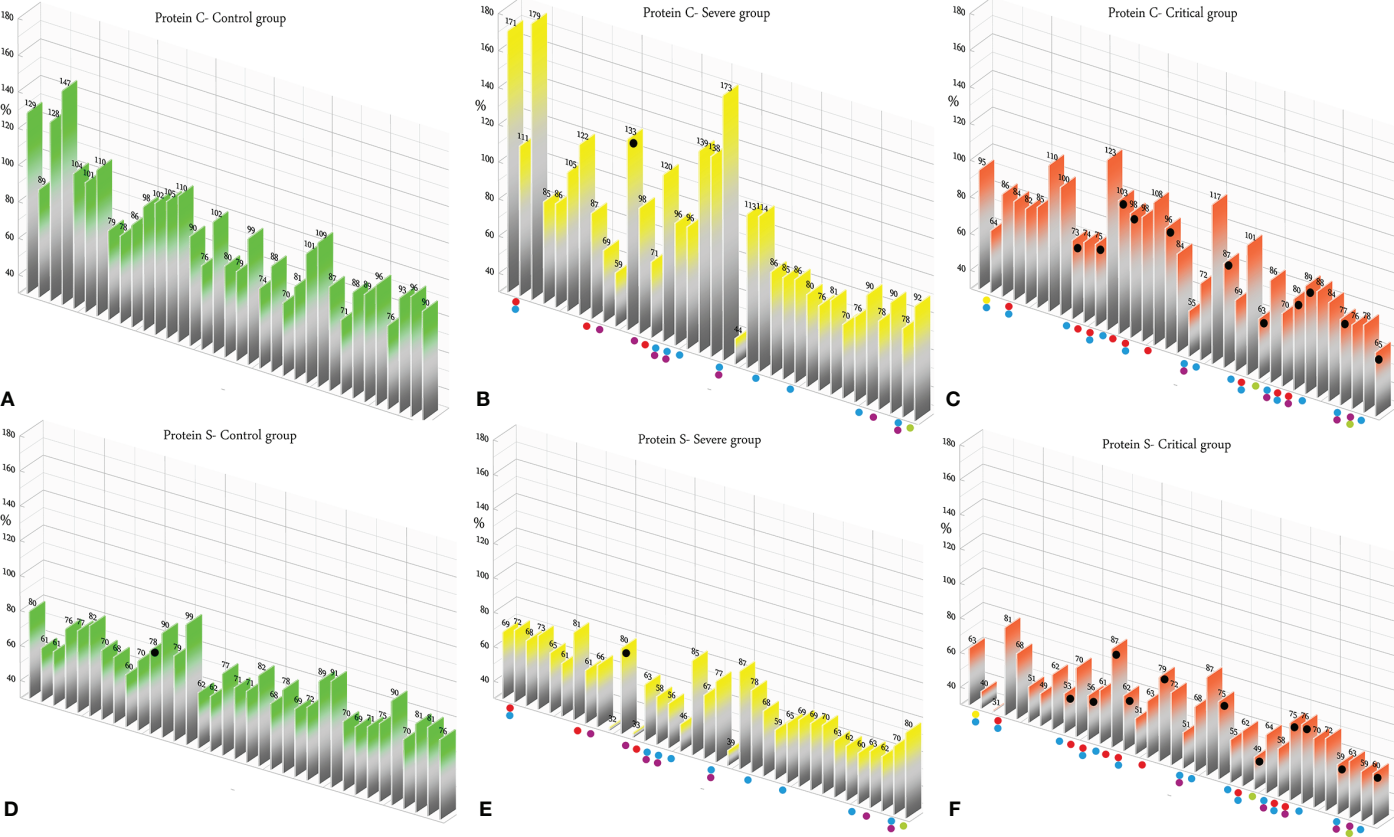
**(A–C)** Protein C test results in the control (CRL, *green*), severe (SVR, *yellow*), and critical (CTL, *red*) groups. **(D–F)** Protein C results in the CRL, SVR, and CTL groups. Expired individuals in the SVR and CTL groups are shown with a *black bullet point*. *Bullet points* in *red*, *blue*, *purple*, *green*, and *yellow* indicate cardiovascular disease, hypertension, diabetes, pulmonary disease, and kidney disease, respectively, in the SVR and CTL groups.

**Figure 5:**
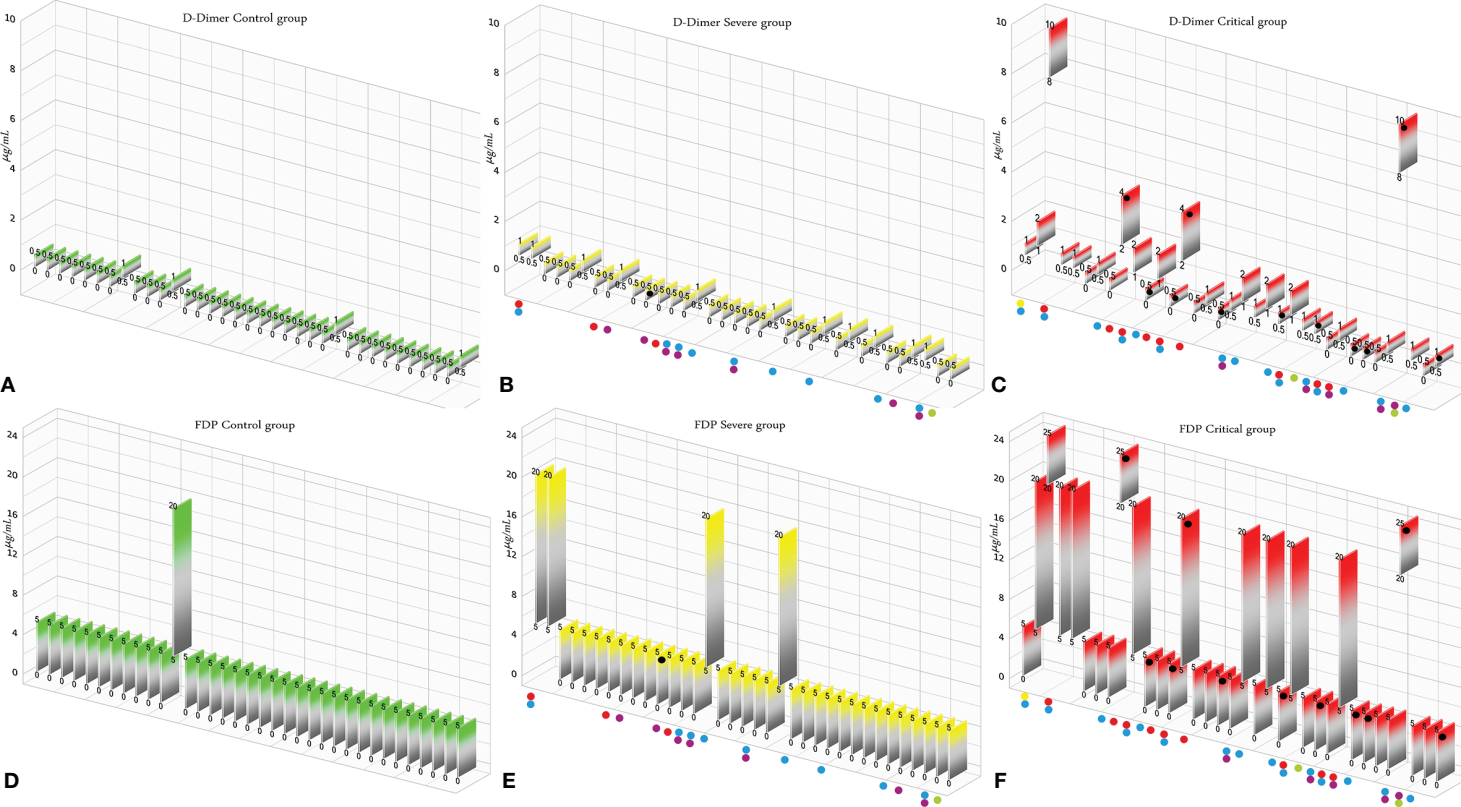
**(A–C)** D-dimer test results in the control (CRL, *green*), severe (SVR, *yellow*), and critical (CTL, *red*) groups. **(D–F)** Fibrin degradation product (FDP) test results in the CRL, SVR, and CTL groups. Expired individuals in the SVR and CTL groups are shown with a *black bullet point*. *Bullet points* in *red*, *blue*, *purple*, *green*, and *yellow* indicate cardiovascular disease, hypertension, diabetes, pulmonary disease, and kidney disease, respectively, in the SVR and CTL groups.

According to the results, there was no association between the age, weight, and BMI of individuals (*p* = 0.92, 0.03, 0.54, respectively). The elevated PT test results have been frequently reported in previous investigations worldwide. **Table 5** summarizes the statistical analysis of the data obtained from the STA Compact® system. Our results showed that while the average PT result in the CRL group was 13.38 ± 0.73 [the international sensitivity index (ISI) of the kit was 1.05; international normalized ratio (INR) = 1.03 ± 0.06], it was significantly elevated in both patient groups, in which the mean PT in the SVR group was 13.85 ± 1.12s (INR = 1.07 ± 0.09) and that in the CTL group was 15.01 ± 1.68s (INR = 1.17 ± 0.14, *p* < 0.001). Notably, the one-way ANOVA results showed a significant difference in the mean PT results between the patient groups (*p* < 0.001). The general trend for the results of the PTT test was similar to that of the PT test, in which the mean results for the test (expressed in seconds) in the CRL group was 36.50 ± 2.64, while it was 37.80 ± 3.73 in the SVR group and 42.92 ± 6.62 in the CTL group. Interestingly, the difference between the two patient groups was statistically significant (*p* < 0.001). For a better presentation of the obtained data, we showed the results of each patient independently. According to **Figures 2A–C**, the PT results showed an increasing trend from the CRL to the SVR and CRL groups. Non-survivors have been marked by black circles. The INR results for each included individual are presented in **Figures 2D, F**. The PTT test results followed a similar trend (minimum in the CRL group and maximum in the CRL group) (**Figures 2G–I**). Analysis of the results for the anti-lupus coagulant test revealed an increasing trend among the groups, in which the average for the controls was 40.76 ± 3.48 s; however, it increased to 47.05 ± 8.25 s in the SVR group and to 49.41 ± 9.24 s in the CTL group. The results for the fibrinogen test provided solid evidence that the fibrinogen levels vary between patients and controls significantly. It is clear that, while the average level of fibrinogen was 283.57 ± 70.51 mg/dl in the CRL group, it went up to 480.29 ± 129.60 mg/dl in the SVR group (*p* < 0.001) and to 537.66 ± 142.68 mg/dl in the CTL group (*p* < 0.001). According to the results of the ATIII test, there was no significant difference among the three studied groups (*p* = 0.321), where the average activity of ATIII in the CRL group was 98.74 ± 10.40%, in SVR group was 99.63 ± 11.56%, and in the CTL group was 95.71 ± 11.96%. The results for each individual for the anti-lupus coagulant test are represented in **Figures 3A–C**. The CRL group had the lowest levels, while the CTL group had the highest levels. Investigation of the fibrinogen levels showed that the CRL group had the lowest levels. The fibrinogen levels were significantly increased in the SVR group; however, the CTL group had the highest levels among all groups (**Figures 3D–F**). The results for ATIII, unlike other tests, revealed that there was no significant difference among the three studied groups (**Figures 3G–I**). Additionally, analysis of the results for protein C showed that the activity of this regulatory protein was 94.31 ± 17.07% in the CRL group, while it increased to 99.34 ± 31.6% in the SVR group. The activity levels of this protein were found to be lower in the CTL group (85.57 ± 15.79%, *p* = 0.04). The ANOVA results showed that the difference between the activity levels of protein C between the CTL and CRL groups was statistically significant, but not to that of the previous tests (*p* = 0.032). Moreover, our result showed that protein S, the cofactor of protein C, had lower activity in the patient groups compared to the CRL group, in which the highest activity was reported in the CRL group (75.03 ± 9.39%), while its activity dropped to 65.06 ± 12.76% in the SVR group. The lowest activity of protein S was observed in the CTL group (62.91 ± 12.32%, *p* < 0.001). According to **Figures 4A–C**, the minimum activity of protein C was observed in the CTL group. A similar trend was observed when we investigated the protein S levels (**Figures 4D–F**). According to the results regarding the D-dimer test, of the 35 individuals in the CRL group, 31 (88.6%) had D-dimer levels <0.5 μg/ml and 4 (11.4%) had D-dimer levels between 0.5 and 1.0 μg/ml. Twenty-four (68.6%) patients in the SVR group were found to have D-dimer levels below 0.5 μg/ml, and 11 (31.4%) had levels between 0.5 and 1.0 μg/ml. In the CTL group, 25 patients had D-dimer levels below 1.0 μg/ml, whereas, only 2 (5.7%) patients had D-dimer levels of 2–4 μg/ml. The same numbers were found to have D-dimer levels over 8 μg/ml [*χ* ^2^(8) = 34.81, *p* = 0.0001] (**Table 6**). Data regarding the D-Dimer test results for each individual are represented in **Figures 5A–C**. There was a significant difference among the three studied groups, in which the CTL group had the highest levels of D-dimer, whereas the CRL group had the lowest levels. The results for FDP also revealed that the majority of healthy controls (34 out of 35) had FDP levels below 5 μg/ml, while only 1 was found to have FDP levels between 5 and 20 μg/ml. In the SVR group, 31 (88.6%) patients had FDP levels below 5 μg/ml and 4 (11.4%) had FDP levels between 5 and 20 μg/ml. In contrast, only 23 patients in the CTL group had FDP levels below 5 μg/ml, and 9 (25.7%) were found to have levels between 5 and 20 μg/ml. There were 3 (8.6%) patients with FDP levels over 20 μg/ml. According to **Figures 5D–F**, the results for each group showed that patients in the CTL group had the highest levels of FDP, while healthy controls had the lowest levels.

**Table 5:** Results of the quantitative tests performed using the STA Compact® system.

Variables	Groups	*N*		Mean		SD		*p*-value	
PT	CTL	35		13.38		0.73		<0.001	
	SVR	35		13.85		1.12		a (CTL/CRT) < 0.001	
	CRL	35		15.01^a^, ^b^		1.68		b (CRL/SVR) < 0.001	
INR	CTL	35		1.03		0.06		<0.001	
	SVR	35		1.07		0.09		a < 0.001	
	CRL	35		1.17^a^, ^b^		0.14		b < 0.001	
PTT	CTL	35		36.50		2.64		<0.001	
	SVR	35		37.80		3.73		a < 0.001	
	CRL	35		42.92^a^, ^b^		6.62		b < 0.001	
Lupus anticoagulant	CTL	35		40.76		3.48		<0.001	
	SVR	35		47.05^a^		8.25		a (SVR/CRL) = 0.002	
	CRL	35		49.41^a^		9.24		a (CTL/CRL) < 0.001	
Fibrinogen	CTL	35		283.57		70.51		<.001	
	SVR	35		480.29^a^		129.60		a (SVR/CRL) < 0.001	
	CRL	35		537.66^a^		142.68		a (CTL/CRL) < 0.001	
ATIII	CTL	35		98.74		10.40		0.321	
	SVR	35		99.63		11.56			
	CRL	35		95.71		11.96			
Protein C	CTL	35		94.31		17.07		0.04	
	SVR	35		99.34		31.60		a (CTL/CRL) = 0.032	
	CRL	35		85.57^a^		15.79			
Protein S	CTL	35		75.03		9.39		<0.001	
	SVR	35		65.06^a^		12.76		a (SVR/CRL) = 0.001	
	CRL	35		62.91^a^		12.32		a (SVR/CRL) < 0.001	

One-way ANOVA, Fisher’s exact test, and chi-square test were used for statistical analysis. A p < 0.05 was considered to be statistically significant.

PT, prothrombin time; INR, international normalized ratio; PTT, partial thromboplastin time; ATIII, antithrombin III; CTL, control group; SVR, severe group; CRL, critical group.

a Significance of the difference with the CTL group, (indicates a P-value of 0.05 or less between the mentioned group and the control group).

b Significance of the difference with the SVR group. (Indicates a P-value of 0.05 or less between the mentioned group and the severe group).

**Table 6:** Results of the semi-quantitative tests including D-dimer and fibrin degradation products (FDPs).

			CRL	SVR	CTL	Total
D-dimer	<0.5	Count	31	24	11	66
		% within group	88.6	68.6	31.4	62.9
	≥0.5 to <1	Count	4	11	14	29
		% within group	11.4	31.4	40.0	27.6
	≥1 to <2	Count	0	0	6	6
		% within group	0.0	0.0	17.1	5.7
	≥2 to <4	Count	0	0	2	2
		% within group	0.0	0.0	5.7	1.9
	≥4 to <8	Count	0	0	0	0
		% within group	0.0	0.0	0.0	0.0
	≥8	Count	0	0	2	2
		% within group	0.0	0.0	5.7	1.9
Total		Count	35	35	35	105
		% within group	100.0	100.0	100.0	100.0
*χ* ^2^(8) = 34.81^a^, *p* = 0.0001						
FDP	<5	Count	34	31	23	88
		% within group	97.1	88.6	65.7	83.8
	≥5 to <20	Count	1	4	9	14
		% within group	2.9	11.4	25.7	13.3
	≥20	Count	0	0	3	3
		% within group	0.0	0.0	8.6	2.9
Total		Count	35	35	35	105
		% within group	100.0	100.0	100.0	100.0
*χ* ^2^(4) = 15.205^b^, *p* = 0.004						

The measurement unit for both tests was micrograms per milliliter.

CTL, control group; SVR, severe group; CRL, critical group.

a Nine cells (60.0%) have expected count less than 5. The minimum expected count is 0.67.

b Six cells (66.7%) have expected count less than 5. The minimum expected count is 1.00.

### Analysis of the Results of Deceased Individuals in the Critical and Severe Groups

We analyzed the data for the deceased individuals (11 out of 35 in the CTL group) and compared them with those of survivors in the same group in order to obtain a better understanding of the impact of abnormal coagulation test results on the fate of the patients. We marked these patients with black bullet points in **Figures 2**–**5**. According to **Figures 2C, F, I** and **Table 7**, the expired individuals in the CTL group had higher mean values for PT, INR, and PTT when compared to all individuals in the CTL group. Additionally, according to **Figures 3C, F, I** and **Table 7**, the expired individuals in the CTL group had higher mean values for LAC, but lower values for the ATIII test. They had slightly lower mean values in the fibrinogen test than the rest of the CTL group. Moreover, according to **Figures 4C, F**, the expired individuals had lower mean values of protein C, but higher protein S, when compared to the mean values in the CTL group. Finally, according to **Figures 5C, F**, individuals who expired were among those with the highest values both in the D-dimer test [≥8 (*n* = 1), 2< to ≥4 (*n* = 2)] and in the FDP test [≥20 (*n* = 2), ≥5 to <20 (*n* = 1)].

**Table 7:** Comparison of the mean values of coagulation tests between expired individuals and all individuals in the critical (CTL) group.

Test	CTL (expired)	CTL (all)
PT	15.53 (median = 15.4, SD = 1.38)	15.01
INR	1.21 (median = 1.2, SD = 0.11)	1.17
PTT	46.85 (median = 48.40, SD = 7.34)	42.92
LAC	53.21 (median = 50.80, SD = 10.30)	49.41
Fibrinogen	533.27 (median = 587, SD = 162.71)	537.66
ATIII	92.63 (median = 94, SD = 10.55)	95.71
Protein C	82.36 (median = 80, SD = 13.32)	85.57
Protein S	66.45 (median = 62, SD = 12.36)	62.91
D-dimer	<0.5 (*n* = 5), 0.5< to ≥1 (*n* = 3) <2 to ≥4 (*n* = 2), ≥8 (*n* = 1)	
FDP	<5 (*n* = 8), ≥5 to <20 (*n* = 1), ≥20 (*n* = 2)	

PT, prothrombin time; INR, international normalized ratio; PTT, partial thromboplastin time; LAC, lupus anticoagulant; ATIII, antithrombin III; FDP, fibrin degradation product.

## Limitations and Recommendations

In this section, we provide recommendations for further investigations (**Table 8**) and address our limitations. We included 35 individuals in each group. Recruiting more patients will provide more accurate results in prospective studies. The enrollment of more patients provides the opportunity to determine cutoffs and design an alarm panel to be used in ICUs. We also applied a single-sampling strategy; however, monitoring the results by obtaining at least 2–3 samples in the CTL and SVR groups could more effectively monitor the test results and their association with the outcomes. One limitation of this study was the use of latex-based semi-quantitative kits to assess D-dimer and FDPs. The results will be more reliable when both tests are performed using fully automated methods. Although the biofunctions of proteins S and C as biological regulators of factors V and VIII are well documented, we did not assess these two factors.

**Table 8:** Technical recommendations and unmet questions awaiting further investigation in COVID-19-associated coagulopathy.

Further investigations on COVID-19-dependent coagulopathy	Reference
**Technical reccommendations**	
Investigations on the stability of the coagulation factors showed that storage at different temperatures, freezing/thawing, affects the activity of the factors. For instance, a change of over 10% for factor V (FV) was reported when the plasma samples were kept at room temperature only for 2 h. Additionally, factors including FII, FVII, FX, and FXII could be affected if they are kept at room temperature over 48 h. Freezing is an effective approach to store these factors; however, long-term storage affects their activity. In this regard, assessment of the impact of sample storage at −20°C showed that the prothrombin time (PT)/international normalized ratio (INR) and FIX results were unaffected only for a month, while the results of aPTT and FVIII remain unaffected for 15 days. To eliminate this pre-analytic problem, we managed to perform the experiment right at the peak when a large number of patients (both critical or severe) were hospitalized. Using this strategy, we performed our study without the need for freezing the samples.	(41, 42)
A bias may occur when the results of the PT test are represented only in seconds and not using INR. This occurs because each PT kit manufactured has a unique international sensitivity index (ISI) parameter, which is used in the calculation of INR: INR = (patient PT/mean normal PT)^ISI^. We recommend including the INR results along with the PT expressed in seconds or at least mentioning the ISI of the used PT kits. This may be beneficial when comparing merely the PT results of different studies without considering the INR or ISI of the kits.	(43)
Considering the low stability of D-dimer and fibrin degradation products (FDPs) over time, we strongly recommend performing these two tests immediately after plasma separation.	(44)
If the study is aimed to be performed on a high number of individuals or it is not possible to collect samples from all individuals in a short time, in which the plasma samples should be stored until running the tests, we recommend monitoring the effect of storage on samples. For this purpose, several samples with low, normal, and high results for the PT and partial thromboplastin time (PTT) tests can be frozen with other samples in separately labeled microtubes to evaluate the test results every 12 or 24 h by comparing the results with those from plasma samples before freezing.	(45)
Considering that pregnant women with physiological pregnancy have higher levels of D-dimer and fibrinogen, we recommend not including them as controls. Additionally, including them in the patient group may result in exaggerated results.	(46)
Fibrinogen levels may vary widely in several bio/pathologic situations, i.e., rise after menopause, rise in diabetes and hypertension, or decrease in alcoholics. We recommend considering such situations in the questionnaire to simply exclude unfit individuals.	(47)
Considering that PO_2_ pressure is a critical factor in placing patients in the critical (CTL) and severe (SVR) groups and that it may vary during a single day in COVID-19 patients, we suggest placing patients with the lowest values into the CTL group and those with the highest into the SVR group and avoiding placing patients with PO_2_ values near the cutoff.	(48)
**Unmet questions**	
C4b-BP has been reported to regulate proteins C and S. Since our results magnified the role of these two regulatory proteins in COVID-19-dependent coagulopathy, investigation of the association between the activity of proteins C and S and the concentration of C4b-BP can be helpful.	(49, 50)
The links between gene mutation and polymorphisms in coagulation regulatory proteins and coagulation disorders have been reported. Studying the association between the SNPs of proteins C and S and ATIII with the prognosis of COVID-19 in patients with coagulopathy could be beneficial.	(51)
Heparin therapy is widely recommended in patients with COVID-19. Considering that it acts as the cofactor for ATIII to inhibit thrombin and factor Xa, an investigation on the impact of heparin therapy on thrombin time (TT) and factor Xa activity may be an interesting theme for further research.	(8)
Proteins C and S regulate the conversion of V to Va and VIII to VIIIa. We suggest investigating these 6 factors for their possible association with the fate of critically ill patients.	(52)

## Discussion

In the present study, we investigated the dynamic changes in 9 coagulation tests on 105 individuals classified into CTL, SVR, and CRL groups. Our study revealed significant aberrant coagulation changes among the studied groups in 4 aspects: extrinsic and intrinsic pathways, fibrinolysis, and the regulatory factors. Our results were consistent with those of the majority of previously published papers. A brief literature review for all tests with consistency levels is represented in **Table 1**. The CTL group had higher PT test (therefore INR) results when compared to the SVR and CRL groups, indicating a disruption in the extrinsic coagulation pathway. In addition, the prolonged PTT results in the CTL group and also similar results in the LAC test showed that not only the extrinsic pathway but even the intrinsic pathway was dysregulated. It should be considered that, in critically ill patients, lupus anticoagulant could be positive. An elevated fibrinogen level was one of the main findings in COVID-19-associated coagulopathy. We showed that there was a significant difference in the fibrinogen levels among the three groups and that the CTL group had the highest levels. It can be used as a common biomarker to predict the severity of the disease; however, the analysis of fibrinogen levels in deceased patients in the CTL group with the whole group showed that it had no significance in predicting death. Investigation of ATIII revealed that its activity was not significantly interrupted in COVID-19 patients (*p* = 0.321). However, proteins C and S, the other regulatory proteins, showed a significant decrease in their activity levels (*p* = 0.04 and *p* < 0.001, respectively). The difference between the reported *p*-values for these proteins was probably due to the low number of individuals recruited in each group; increasing the sample size will provide more accurate data. Considering that proteins C and S regulate the conversion of factors V and VIII to their active forms, we conclude that the disruption of homeostasis in protein C (and S) regulating the conversion of factors V and VIII to their active form could be a mechanism for COVID-19-associated coagulopathy. The fibrinolysis pathway was also affected in the presence of SARS-COV-2, in which the production of FDPs, mainly D-dimer, was accelerated, and according to our results, deceased patients were found to have significantly higher FDP and D-dimer levels when compared to survivors. The majority of coagulation factors are produced in the liver; to prevent the effects of hepatopathy on the levels of the coagulation factors and the corresponding tests, we enrolled normal controls and patients whose liver function tests were normal. Interestingly, factors including FVIII and vWF (which act as markers of endothelial activation) (53) were produced in the endothelial cells. Investigation of the levels of these factors in COVID-19 patients revealed that their levels increased and may correlate with poorer prognosis (54–56). We showed that D-dimer, fibrinogen, PT, PTT, LAC, protein S, FDPs, and protein C (ordered according to their *p*-values) could effectively be used in the prognosis of the severity of the disease and that disruptions in proteins C and S regulating the conversion of factors V and VIII to their active form may interfere the homeostasis of the coagulation system.

## Data Availability Statement

The original contributions presented in the study are included in the article/supplementary material. Further inquiries can be directed to the corresponding author.

## Ethics Statement

The studies involving human participants were reviewed and approved by the Ethics Committee of Urmia Medical University (IR.UMSU.REC.1399.264). The patients/participants provided written informed consent to participate in this study.

## Author Contributions

DEAK designed the study, performed the tests, generated the figures, and prepared the manuscript. YR contributed to performing semiquantitative tests. RA and RJ supervised the recruitment of patients and the control group. JR analyzed the data. MM performed sample preparation. AA supervised quality control. RN and FR collected clinical data. VS-I supervised the research progression. All authors contributed to the article and approved the submitted version.

## Funding

This study was supported by the Cellular and Molecular Research Center, Cellular and Molecular Medicine Institute, Urmia University of Medical Sciences (fund no. 1399.264).

## Conflict of Interest

The authors declare that the research was conducted in the absence of any commercial or financial relationships that could be construed as a potential conflict of interest.

## Publisher’s Note

All claims expressed in this article are solely those of the authors and do not necessarily represent those of their affiliated organizations, or those of the publisher, the editors and the reviewers. Any product that may be evaluated in this article, or claim that may be made by its manufacturer, is not guaranteed or endorsed by the publisher.

## Abbreviations

COVID-19, coronavirus disease 2019; PT, prothrombin time; aPTT, activated partial thromboplastin time; ATIII, antithrombin III; LAC, lupus anticoagulant; ISI, international sensitivity index; INR, international normalized ratio; ICU, Intensive care unit; CRL group, control group; SVR group, severe group; CTL group, critical group.
